# A Decade of Data Protection for Innovative Drugs in Canada: Issues, Limitations, and Time for a Reassessment

**DOI:** 10.1089/blr.2016.29030.mk

**Published:** 2016-12-01

**Authors:** Megan Kendall, Declan Hamill

**Keywords:** data protection, innovative drugs, Canada, medicines

## Abstract

Drug regulators in Canada and in other nations require innovative pharmaceutical companies to submit undisclosed clinical or other data as a condition of approving the marketing of new pharmaceutical products—the origination of which involves considerable effort and investment. Data protection regulations were enacted in Canada in 2006, which—to some extent—closed a loophole in intellectual property law that had previously left innovative companies with no effective data protection for their clinical data. Although the regulations were intended to clarify and effectively implement Canada's international treaty obligations in the spirit of innovation, a review of Canada's first decade of effective data protection shows that Health Canada and Canadian courts have interpreted the scope of data protection for innovative drugs in a narrow manner that undermines and is inconsistent with the intent of the regulations. As the 10-year anniversary of data protection in Canada is this year (2016), this article demonstrates the need to advance Canada's data protection regime into one that consistently contributes to the promotion of investment in pharmaceutical research and development, to the mutual advantage of innovators and patients, in a manner conducive to the social and economic welfare of Canadians.

## 1. What is the Purpose of Data Protection for Innovative Drugs?

When an innovator company is seeking regulatory approval for a new innovative drug in Canada, it is required by law to submit the results of its clinical testing data to Health Canada so that the product's safety and efficacy may be verified. Data protection provides a limited duration of time during which only the owner or generator of this preclinical and clinical trial data can use it to obtain marketing authorization for biopharmaceutical and pharmaceutical products.

When a suitable level of data protection is afforded, this process becomes one essential way in which innovation is promoted in the biopharmaceutical sector. This is especially important given that, although a patent lasts for 20 years in Canada, it takes on average 10 years or more for a new medicine or vaccine to go through all the trial and approval stages—often leaving companies less than 10 years to recover their investment.^1^ Developing complex treatments to fight such illnesses as diabetes, heart disease, or cancer is extremely expensive, time consuming, and risky: taking the situation in the United States as an example, the economic cost to develop, win marketing approval, and conduct post-approval research and development (R&D) for a new drug as required by the U.S. Food and Drug Administration averages between US$2.6 billion and US$2.9 billion,^2^ while there is a less than 12% probability that a drug entering clinical testing will eventually be approved for marketing.^3^ These risks are growing alongside Canada's rapidly evolving biopharmaceutical market: some of the most promising new therapeutic products are biologic drugs (products derived from living sources), which on average do not recover their R&D costs until 17 years after they start being sold.^4^ It is a similar situation with “orphan drugs,” which are medications used to treat rare diseases, typically affecting fewer than five in 10,000 people. The vast majority of the diseases for which orphan drugs would be beneficial are linked to genetic factors, so development of new treatments is very costly and the market is very small, making cost recovery more difficult than for drugs with larger potential patient populations. The protection of undisclosed test data submitted for the approval of innovative drugs seeks to balance these risks by acting as an incentive for local and foreign pharmaceutical companies to make the enormous R&D investments necessary for new medicines and vaccines, while also inciting homegrown R&D.

In Canada, data protection regulations were enacted in 2006 under the *Food and Drug Regulations*^5^ (the “Regulations”), which—to some extent—closed a loophole in intellectual property (IP) law that left innovative companies with no effective data protection in Canada for their clinical data. These were enacted at the same time as amendments to the *Patented Medicines (Notice of Compliance) Regulations*, which together—according to the Government of Canada—were meant “to act as a balanced set of measures, designed to work together to stabilize Canada's intellectual property protection for drugs by ensuring a minimum period of protection and maintaining a reasonable ceiling on the maximum protection available.”^6^ Under the previous Regulations, the data protection exclusivity period arose only when the Minister examined and relied on an innovator's undisclosed data in order to grant a notice of compliance to a generic manufacturer. In practice however, the Minister would typically assess the bioequivalence of a generic relying on the innovator's product, but without relying on the innovator's data. According to the Regulatory Impact Analysis Statement (RIAS), which accompanies but does not form part of the regulations, the amendments were introduced “to clarify that the aforementioned reliance [on the innovator's product] will give rise to an exclusivity period.”^7^

The Regulations, at section C.08.004.1, entitle innovative drugs to an eight-year term of data protection (plus a possible additional six months for submissions that include pediatric studies), and prevent a second-entry manufacturer from filing a submission for a copy of that innovative drug for the first six years of the eight-year period. Data protection is provided if and when a drug is given approval to be added to Health Canada's Register of Innovative Drugs,^8^ at the time a Notice of Compliance is issued to a manufacturer following the successful review of a submission for a new drug. For greater clarity, section C.08.004.1(3) of the Regulations is reproduced below:

(3) If a manufacturer seeks a notice of compliance for a new drug on the basis of a direct or indirect comparison between the new drug and an innovative drug,

(a) the manufacturer may not file a new drug submission, a supplement to a new drug submission, an abbreviated new drug submission or a supplement to an abbreviated new drug submission in respect of the new drug before the end of a period of six years after the day on which the first notice of compliance was issued to the innovator in respect of the innovative drug; and

(b) the Minister shall not approve that submission or supplement and shall not issue a notice of compliance in respect of the new drug before the end of a period of eight years after the day on which the first notice of compliance was issued to the innovator in respect of the innovative drug.

Data protection is not an extension of patent rights. In fact, upon its conclusion after eight years, it is frequently surpassed by patent rights. But if a patent period has expired, or there is no patent on a product (possibly as a result of being judicially invalidated), data protection will act independently to prevent entry of a competitor product on the market until the period of data protection ends (although a competitor is free to develop a product and conduct its own testing to seek authorization).

## 2. What are the Issues in Canada with Respect to Data Protection?

By enacting effective data protection regulations in 2006, the federal government has sent a strong signal to the world that Canada is serious about attracting R&D in a globally competitive market. Unfortunately, as the 10-year anniversary of the Regulations is upon us, it has become clear that both Health Canada and Canadian courts have interpreted data protection for innovative drugs in such a narrow manner so as to reduce the intent of data protection as an incentive for innovation.^9^

Data protection for innovative drugs is not a Canada-specific legal requirement, but rather is found in IP systems around the world. As a demonstration of the role of data protection in fostering innovation, as well as the desire of trading partners to level the playing field in this regard, data protection has been negotiated with the IP obligations under several of Canada's most important international trade treaties. Most recently, data protection was a contested topic during Canada's free trade negotiations with the European Union under the Comprehensive Economic and Trade Agreement, as well as among the 12 Pacific Rim negotiating parties to the Trans-Pacific Partnership. And foundationally speaking, the data protection regulations explicitly state that they apply to the implementation of data protection obligations under both the North American Free Trade Agreement (NAFTA) and the Agreement on Trade-Related Aspects of Intellectual Property Rights (TRIPS):

The purpose of this section is to implement Article 1711 of the North American Free Trade Agreement, as defined in the definition Agreement in subsection 2(1) of the North American Free Trade Agreement Implementation Act, and paragraph 3 of Article 39 of the Agreement on Trade-Related Aspects of Intellectual Property Rights set out in Annex 1C to the Agreement Establishing the World Trade Organization, as defined in the definition Agreement in subsection 2(1) of the World Trade Organization Agreement Implementation Act.^10^

Therefore, the maximum eight and a half years of data protection available in the Canadian system must be considered in light of the maximum term of eight and a half years in the United States for chemical products and 12 years for biologic products, the maximum of 11 years in the European Union, and the up to eight years equivalent protection (with potential additional time available) in Japan.

Canada's eight-year base term of data protection applies to all drugs, including biologics and orphan drugs. On the other hand, both the United States and the European Union add several years of data protection for orphan drugs to incentivize their R&D, while comparable benefits are on offer in Japan, Singapore, South Korea, and Taiwan. Canada, by contrast, currently offers no exclusivity incentives to specifically encourage orphan drug development, and the previous government's promised Orphan Drug Regulatory Framework stated that the existing eight-year data protection provisions under section C.08.004.1 of the Regulations will continue to apply to a market authorization issued for an orphan drug.^11^ However, data protection will not apply at all to known drugs that are developed for orphan indications, since such drugs would not be deemed to be “innovative.”

This dilution of data protection's value has been intensified by several Federal Court of Canada (FCC) decisions that have upheld a strict interpretation of the “previously approved” and “variation” aspects of the definition of “innovative drug.” Under the Regulations, an “innovative drug,” by definition, ”means a drug that contains a medicinal ingredient not previously approved in a drug by the Minister [of Health] and that is not a variation of a previously approved medicinal ingredient such as a salt, ester, enantiomer, solvate or polymorph.”^12^ The regulatory provisions on their face are consistent with Canada's treaty obligations, but their judicial interpretation, particularly of two terms—“previously approved” and “variation of a previously approved drug”—has been arguably inconsistent with the policy underpinnings and intent of those data protection obligations.

*Epicept* was the earliest case in which the FCC considered the meaning of “innovative drug” in light of a “previously approved” drug.^13^ In that case, Epicept challenged the Minister's decision that said that the drug CEPLENE was not an “innovative” drug as required by the data protection regulations in order to be afforded data protection. The basis for this decision was that, while CEPLENE was a “new drug,” developed for a new oncology indication based on a full package of clinical data, its active ingredient (histamine dihydrochloride) had been previously approved by the Minister by way of its inclusion in another drug—in that case, for homeopathic uses. Notwithstanding the fact that homeopathic drugs are not approved in the same way as therapeutics and are subject to a completely different Health Canada regulatory process, the Federal Court engaged the “previously approved” criterion of the Regulations and upheld the Minister's interpretation: a “new drug” for regulatory approval purposes is not necessarily “innovative” as that term is framed in the Regulations, and will be ineligible for data protection if its active ingredient has previously been given approval by way of another drug.

In the *Celgene* case, the courts again focused their attention to the “previously approved” aspect of an “innovative drug.”^14^ Celgene sought judicial review of the Minister's decision to refuse to list THALOMID on the Register of Innovative Drugs on the basis that its ingredient (thalidomide) had been previously approved in a drug in Canada decades earlier, despite the fact that the earlier drug had another clinical usage and was subsequently withdrawn from the market for being unsafe. Celgene claimed its drug was in fact an “innovative” drug in view of the 1960s withdrawal, which it claimed made the previous approval a nullity. Moreover, Celgene pleaded it had generated its own clinical trial data for a completely different disease condition than the original product. The Federal Court, at the first instance held that the purpose and intent of the Regulations was to encourage innovation and the development of new drugs, and that THALOMID was an “innovative drug” and should benefit from eight years of data protection. Deciding against the innovator in this case and setting a precedent for many who will follow, the Federal Court of Appeal (FCA) sided with the Minister's interpretation that the word “approved” is qualified by the adverb “previously,” and therefore must include a medicinal ingredient that previously satisfied Canadian regulatory requirements before its approval was revoked.^15^

In the *Takeda* case, the FCA considered the basis for exclusion from data protection relating to the term “variations.”^16^ Takeda argued that the Minister erred in denying data protection for its drug DEXILANT, an enantiomer (mirror image) of a previously approved drug. In a split decision, the Court denied Takeda's request for data protection, holding that the five listed examples of variations in the definition of “innovative drug” (a salt, ester, enantiomer, solvate, or polymorph) will always be a mere “variation” and therefore not an innovative drug. Justice Stratas, in a strong dissent, rejected a literal reading of the definition of “innovative drug” in the Regulations, finding that the term “such as” interjected uncertainty as to whether the five categories of substances were to be automatically considered variations. In addition, Justice Stratas found the decision ran contrary to the Minister previously having granted data protection to certain drugs which contain medicinal ingredients that are esters or enantiomers, such as TORISEL, PRECEDEX, and AVAMYS.^17^ Justice Stratas used the Minister's qualification of these three drugs as “innovative drugs” as evidence that the variation element is the controlling idea of the Regulations as per their RIAS, which states that “variations” are excluded from the definition of “innovative drugs” in order to prevent “the granting of an additional eight years of protection where an innovator seeks approval for a minor change to a drug.”^18^ The majority also referred to the RIAS, but interpreted it differently, to mean that the nature of previously submitted clinical data should only be considered for substances other than the enumerated variations when deciding whether or not data protection should be granted.^19^

This restrictive majority interpretation of “variations” has since been applied by the FCC in support of the Minister's refusal to grant data protection for at least one ostensibly innovative drug. In *Photocure*, for example, the FCC relied heavily on *Takeda* in agreeing with the Minister's refusal to grant “innovative drug” status to CYSVIEW due to the view that its active ingredient HAL HCl was a variation of ALA HCl.^20^ Notwithstanding that the FCA in *Takeda* held that the standard of review of the Minister's interpretation of the data protection regulations is generally correctness, the FCC applied a standard of reasonableness in concluding that the Minister's decision was one that could withstand a “somewhat probing examination.” This is a low threshold given the complexity of the science behind innovative drugs.

In addition, many—including innovators in the above case law—argue that Canada's interpretation of “innovative drugs” in the Regulations departs from the purpose of long-standing international treaty obligations. For example, under TRIPS, Canada is obligated to protect data against “unfair commercial use.”^21^ In a purposive interpretation of the Regulations, the fairness of a competitor's use of data therefore has to be considered. Likewise, Canada and its NAFTA partners agreed—should they require the submission of data to determine a product's safety and effectiveness—to “protect against disclosure of the data of persons making such submissions, where the origination of such data involves considerable effort, except where the disclosure is necessary to protect the public or unless steps are taken to ensure that the data is protected against unfair commercial use.”^22^ As a result, in *Celgene*, the argument underlying the innovator's position was that it took the time and expense of demonstrating THALOMID's newfound safety in a new application, thereby fulfilling the criteria set forth by these treaties for protection against the unfair commercial use of such undisclosed data, the origination of which involved considerable effort.^23^ Still, the FCA interpreted the scope of application of TRIPS and NAFTA data protection as being exclusionary to medicinal ingredients historically approved even if later withdrawn. Again, in *Takeda*, while acknowledging that the data protection regulations were intended to implement Canada's obligations under NAFTA and TRIPS, the court's majority held that it was open to the Governor in Council to decide, as a matter of policy, that the five categories of substances listed in the definition of an “innovative drug” were not sufficiently different to be “new chemical entities.” And yet recently, when the FCC interpreted the scope of application of data protection rather than the definition of “innovative drug,” it based its interpretation in part on the purpose of the data protection provisions: namely, meeting Canada's treaty obligations and its commitments to protecting innovators from unfair commercial use of undisclosed data.^24^

Regardless of the degree to which treaties such as NAFTA and TRIPS allow for local adaptation upon implementation, it is settled law in Canada that when interpreting statutes, international law should be considered and applied—to the extent that the words of the statute allow—without creating conflict.^25^ The Supreme Court of Canada (SCC) has additionally affirmed that, in deciding between possible interpretations, courts will avoid a construction that would place Canada in breach of those obligations.^26^

## 3. How Have Pharmaceutical Innovators Been Impacted?

The industry experience in seeking judicial review of Health Canada's decisions regarding what is or is not an “innovative drug” in the courts has been both disappointing and unsuccessful to date. The dissenting opinion in *Takeda* specifies several negative outcomes of the current judicial precedent for the pharmaceutical industry, including the undermining of incentives for the development of beneficial new drugs.^27^ In that particular case, Justice Stratas observed that, “if enantiomers are automatically excluded, then […] the innovators of other drugs whose medical ingredients are enantiomers that give rise to greater safety and efficacy […] would not receive data protection.”^28^ The sum of other literal and conservative court decisions have left a number of innovators without data protection, thereby potentially inhibiting the research, discovery, and development of new, safe, and efficacious drugs.^29^

The industry impact of decisions to deny data protection was also made evident when Epicept asked the FC for confidentiality order to prevent the public disclosure of the Minister's initial decision to deny CEPLENE data protection.^30^ Epicept submitted that it would be harmed by the “head start” its competitors would enjoy to prepare regulatory submissions to enter the market themselves over the course of the judicial review. And again later, Epicept's appeal to the FCA was dismissed as moot since the company had already chosen to withdraw its submission to Health Canada for approval to market CEPLENE in Canada.^31^

Such was the line of thinking in a recent case challenging the Minister's decision to refuse data protection. This time, Horizon challenged the Minister's refusal to grant data protection for its RAVICTI drug on the basis that it was an ester variation of a previously approved drug. Horizon successfully moved to stay the issuance of its own approval pending judicial review of this decision.^32^ Horizon stated that it would have no choice but to withdraw its drug from the Canadian approval process if it is not granted data protection—not out of spite, but in order to prevent generic competitors from using the information therein contained and entering the market immediately upon the expiry of its Canadian patent.^33^ In doing so, the potential impacts on industry and society were made clear: Horizon would suffer non-compensable losses and Canadian patients suffering from urea cycle disorders would be denied access to a life-changing and life-saving drug. After the Federal Court reverted the decision back to the Minister for reconsideration, RAVICTI was granted data protection for reasons not made public.

Extending beyond industry, the net effect in the cases here discussed has been the denial of data protection for drugs used for such purposes as the treatment and detention of life-threatening diseases, including cancer and leprosy and other related conditions,^34^ for remission maintenance in acute myeloid leukemia,^35^ and for the treatment of gastroesophageal reflux disease (a common, recurring problem affecting 10%–20% of the Canadian population).^36^ When paired with the absence of data protection incentives for biologic and orphan drugs, it is unclear if innovators will have sufficient incentives to seek product approvals in Canada so that they may bring new treatments to Canadian patients afflicted with rare disease conditions. Drugs at issue in the above-noted case law, namely, CEPLENE, THALOMID, and RAVICTI, are but several examples of orphan drugs—and each has been granted orphan drug status and associated benefits in other jurisdictions.^37^

In addition, the interpretation of the data protection requirements by Canadian courts harms the Canadian marketplace by putting Canada at odds with its interpretation in other jurisdictions. The approach taken by Health Canada and Canadian courts regarding what is and is not an innovative drug is far more restrictive than the approaches regarding the scope of data protection taken elsewhere. For example, in contrast with the interpretation handed down in *Takeda*, the domestic laws of various foreign jurisdictions with legal traditions similar to Canada's (*e.g.*, Europe, the United States, Australia, and New Zealand) do not withhold data protection from drugs merely because their medicinal ingredients are enantiomers of previously approved drugs.^38^ This divergence conflicts with the realities faced by biopharmaceutical innovators, in that they often have to go through the marketing authorization process in multiple jurisdictions in an attempt to offset the extremely high costs associated with developing new drugs. By deviating from the practices of its major trading partners, Canada's status as a legislative outlier may result in certain innovative medicines never being submitted to Health Canada so that they may be made available for patients. In the absence of data protection, there is a less compelling business case to do so, given that a substantial investment will be made in gaining approval only to have a generic competitor enter the market shortly after approval. This scenario is also problematic for generic drug manufacturers, who rely upon the clinical test data originally provided to Health Canada by innovators for their own more expedited drug approval processes.

A more favorable development over the last 10 years has been the determination that drugs that have been approved by way of emergency treatment under Health Canada's Special Access Programme (SAP) shall not be deemed to have been “previously approved” as per the data protection regulations. This was the conclusion in *Teva*, where the validity of ELOXATIN's data protection was maintained despite being challenged by a generic that had earlier been approved for emergency purposes under SAP in the absence of data and studies demonstrating its drug's safety and efficacy.^39^ Data protection for ELOXATIN was again maintained in *Hospira*, where the FC determined that data protection regulations are triggered even when post-filing amendments to a marketing application make direct or indirect comparison to an innovative drug.^40^

## 4. What are the Potential Solutions?

Pharmaceutical innovators seeking regulatory approval of innovative drugs in Canada should be aware of the restrictive approach generated by a decade of administrative decisions supported by case law, while monitoring potential avenues for change. Given that this dynamic has been largely created by the case law, the conventional remedy is for a higher court to fix the problem. Despite the FCA's split decision in *Takeda*, the SCC refused Takeda's application for leave.^41^ At this time, it remains unclear if or when another data protection case might reach the SCC.

Although IP has been addressed as a trade barrier in Canada's recent trade agreements and negotiations, data protection has only been considered in terms of the length of time innovative drugs must be protected. While the base term of data protection is clearly important, by failing to harmonize Canada's rules that classify a drug as an innovative drug, Canadian innovators are limited in their ability to compete on a more even playing field with international pharmaceutical firms under these trade deals. Further, the current interpretations discourage foreign investors from placing pharmaceutical research and development funds in Canada. Most importantly, the narrow interpretation of what products qualify for data protection is detrimental for Canadian patients, who may not have access to innovative drugs available to patients in other nations. A change of interpretation would require another company to make a challenge or for the Minister to effect regulatory changes to the interpretations.

Accordingly, the data protection provisions of the Regulations should be amended so as to overturn the restrictive case law that has emerged from the Federal Court level. The legislative authority for data protection underlies the current Regulations, and as Parliament stipulated in section 30(3) of the *Food and Drugs Act*,^42^ the Governor in Council may make regulations:

that the Governor in Council considers necessary for the purpose of implementing, in relation to drugs, Article 1711 of the North American Free Trade Agreement or paragraph 3 of Article 39 of the Agreement on Trade-related Aspects of Intellectual Property Rights set out in Annex 1C to the WTO Agreement.

Revisiting the definition employed in Canada's data protection regulations, an “innovative drug means a drug that contains a medicinal ingredient not previously approved in a drug by the Minister and that is not a variation of a previously approved medicinal ingredient such as a salt, ester, enantiomer, solvate or polymorph.”^43^ To avoid case law limiting the interpretation of this definition, further specificity must be provided against the subjectivity currently afforded by the term “variation.” Such precise language was avoided in Australia, for example, which legislatively mandates data protection for “new goods” when, simply, no goods consisting of or containing the same active ingredient were (or had ever been) included in its national Register of Therapeutic Drugs.^44^ New Zealand's data protection legislation similarly limits its applicability to an innovative drug containing an active ingredient that had not previously been referred to in any other marketing application as an active ingredient of a medicine.^45^

Another approach would be to remove the uncertain language “such as” that is used in association with very certain scientific descriptors: salt, ester, solvate, and polymorph. The United States offers another, and arguably clearer, model, where data protection applies only to the first approval of a drug product that does not contain an active moiety that has been previously approved.^46^ The regulation very clearly defines “active moiety” as the molecule or ion, excluding those appended portions of the molecule that cause the drug to be an ester, salt (including a salt with hydrogen or coordination bonds), or other noncovalent derivative (such as a complex, chelate, or clathrate) of the molecule, responsible for the physiological or pharmacological action of the drug substance.^47^ The laws of these three countries are additionally helpful in their illustration of how Canada should avoid regulatory language that will automatically withhold data protection from drugs merely because their medicinal ingredients are enantiomers.

The amendments should be accompanied by a Regulatory Impact Analysis Statement that clearly describes the underlying intent: to protect innovation and to encourage companies to submit products to Health Canada for safety approval in order to improve access for Canadian patients to new medications. This type of clarification is of particular importance as the standard of review to be applied by the courts to the Minister's interpretation of the data protection provisions is correctness.^48^ Under correctness review, courts are required to interpret for themselves the language used in legislation and regulations (including with the aid of a RIAS), and the Minister's interpretation of the definition of “innovative drug” in other cases is not determinative of the accuracy of the interpretation.

Alternatively, it may be sufficient for Health Canada to clarify its *Guidance Document: Data Protection under C.08.004.1 of the Food and Drug Regulations*,^49^ which currently offers little practical guidance on the terms as written. While these guidelines have been referenced in Federal Court decisions involving data protection,^50^ they are solely administrative aids.^51^ However, amending the Regulations as set out above would give clarifications the force of law, and ensure that the unfavorable precedents described in this article not be followed in the future—all the while insulating the data protection scheme from simple variants.

For the time being, a narrow approach to the provision of data protection for innovative drugs in Canada remains a significant concern for both domestic and foreign innovators and investors. The interpretative character of this vital set of rights has left innovators to question whether the Canadian system is striking the necessary balance between technological innovation and market sustainability. Until greater clarity is achieved, the overarching question remains: Have Health Canada and the Canadian courts interpreted data protection provisions in a manner that equitably protects innovation, and that encourages the approval of new treatments for Canadian patients?

Canada's data protection regime will mark its tenth anniversary this year in 2016. Having survived several jurisdictional challenges, its validity is no longer in question.^52^ Moreover, it appears that a minimum term of eight years from regulatory approval may be further enshrined as a result of Canada's new generation of trade agreements.^53^ But after nearly a decade of interpretation and jurisprudence, the time has come for an assessment of how the regime has performed, and how it can be improved to better protect innovation in Canada and better encourage the introduction of new treatments for Canadian patients.

## Notes

^1^ J.A. DiMasi, H.G. Grabowski, and R.W. Hansen, *Briefing: Cost of Developing a New Drug—Innovation in the Pharmaceutical Industry: New Estimates of R&D Costs*, Tufts Center for the Study of Drug Development (Nov. 18, 2014), http://csdd.tufts.edu/files/uploads/Tufts_CSDD_briefing_on_RD_cost_study_-_Nov_18,_2014..pdf (last accessed Nov. 4, 2016).

^2^ Id.

^3^ Pharmaceutical Research and Manufacturers of America (PhRMA), *Biopharmaceutical Research & Development: The Process Behind New Medicines*, PhRMA (2016), http://www.phrma.org/sites/default/files/pdf/rd_brochure_022307.pdf (last accessed Nov. 4, 2016).

^4^ John A. Vernon, Alan Bennett, and Joseph H. Golec, *Exploration of Potential Economics of Follow-On Biologics and Implications for Data Exclusivity Periods for Biologics*, 16 BUJ Sci. & Tech. L. 55 (2010).

^5^ *Food and Drug Regulations*, C.R.C., c. 870.

^6^ Canada Gazette Part II: Regulatory Impact Analysis Statement, SOR/DORS/2006-241; 140(21):1495–1502 at 1499 [RIAS].

^7^ *Id.* at 1495–1496.

^8^ The Register of Innovative Drugs is available online: Health Canada, http://www.hc-sc.gc.ca/dhp-mps/prodpharma/applic-demande/regist/reg-innov-dr-eng.php (last accessed May 12, 2016). As of November 4, 2016, the Register of Innovative Drugs lists as having data protection: 234 (active) and 54 (expired) products for human use, and 37 (active) and 12 (expired) products for veterinary use. Among the products for human use, 63 of those active and 21 of those expired were granted a pediatric extension of six months.

^9^ *See, e.g.*, Ted Buckley, Joseph H. Golec, and John A. Vernon, *Data Exclusivity Period Length and Federal Government Savings from Enactment of the Biologics Price Competition and Innovation Act of 2007* (Jan. 26, 2009), http://papers.ssrn.com/sol3/papers.cfm?abstract_id=1333263 (last accessed Nov. 4, 2016); Erkki Liikanen, *Changes to the European Union Pharmaceutical Legislation: Implications of the “Pharma Review” for Biotechnology Companies*, 11(1) J. Commerc. Biotechnol. 38 (2004); Henry Grabowski, *Follow-On Biologics: Data Exclusivity and the Balance Between Innovation and Competition*, 7(6) Nat. Rev. Drug. Discov. 479 (2008); Dana P. Goldman, Darius N. Lakdawalla, *et al*., *The Benefits from Giving Makers of Conventional “Small Molecule” Drugs Longer Exclusivity over Clinical Trial Data*, 30(1) Health Affairs 84 (2011).

^10^ Food and Drug Regulations, *supra* note 5, at C.08.004.1(2). NAFTA, which came into force in 1994, is an agreement signed by Canada, Mexico, and the United States, creating a trilateral rules-based trade bloc in North America. In 1994, Canada became bound by TRIPS, an international agreement administered by the World Trade Organization (WTO), which sets down minimum standards for many forms of intellectual property regulation as applied to nationals of other WTO members.

^11^ Office of Legislative and Regulatory Modernization, Policy, Planning and International Affairs Directorate, Health Products and Food Branch, *Initial Draft Discussion Document for a Canadian Orphan Drug Regulatory Framework*, Orphanet, at J (Ottawa: Government of Canada, 2012), http://www.orpha.net/national/data/CA-EN/www/uploads/Initial-Draft-Discussion-Document-for-A-Canadian-Orphan-Drug--Regulatory-Framework.doc (last accessed Nov. 4, 2016).

^12^ *Food and Drug Regulations, supra* note 5, at C.08.004.1(1).

^13^ *Epicept Corporation v. Canada (Health)*, 2010 F.C. 956 [*Epicept*].

^14^ *Celgene Inc. v. Canada (Health)*, 2012 F.C. 154 [*Celgene*, FC].

^15^ *Canada (Health) v. Celgene Inc*., 2013 F.C.A. 43 [*Celgene*, FCA].

^16^ *Takeda Canada Inc. v. Canada (Health)*, 2013 F.C.A. 13 [*Takeda*].

^17^ *Id.* at 57–59.

^18^ RIAS, *supra* note 6, at 1496, as cited in *Takeda*, *supra* note 16, at 63.

^19^ *Takeda*, *supra* note 16, at 124–127.

^20^ *Photocure ASA v. Canada (Health)*, 2015 F.C. 959.

^21^ *Agreement on Trade-Related Aspects of Intellectual Property Rights, being Annex 1C of the Marrakesh Agreement Establishing the World Trade Organization*, Apr. 15, 1994, 1869 U.N.T.S. 1994, at art. 39(2).

^22^ *North American Free Trade Agreement Between the Government of Canada, the Government of Mexico and the Government of the United States*, Dec. 17, 1992, Can. T.S. 1994 No. 2, 32 I.L.M. 289 (entered into force Jan. 1 1994), at art. 1711(5).

^23^ *See, e.g*., *Celgene*, FCA*, supra* note 15, at 24.

^24^ *Hospira Healthcare Corporation v. Canada (Health)*, 2015 F.C. 1205 [*Hospira*], at 83.

^25^ *Daniels v. White*, [1968] S.C.R. 517; *Ordon Estate v. Grail*, [1998] 3 S.C.R. 437.

^26^ *R v. Hape*, [2007] 2 S.C.R. 292, at 53.

^27^ *Takeda*, *supra* note 16, at 98.

^28^ *Takeda*, *supra* note 16, at 98.

^29^ *Takeda*, *supra* note 16, at 102.

^30^ *Epicept, supra* note 13.

^31^ *Epicept Corporation v. Canada (Health)*, 2011 F.C.A. 209.

^32^ *Horizon Pharma plc v. Canada (Health)*, 2015 F.C. 744.

^33^ *Id*. at 2.

^34^ The drug THALOMID, as in *Celgene*; and the drug CYSVIEW, as in *Photocure*.

^35^ The drug CEPLENE, as in *Epicept*.

^36^ The drug DEXILANT, as in *Takeda*.

^37^ CEPLENE has been granted orphan drug status for the treatment of acute myeloid leukemia by the European Medicines Agency and the U.S. Food and Drug Administration [Immune Pharmaceuticals, http://www.immunepharma.com/product-portfolio/ceplene/, last accessed Nov. 4, 2016]; THALOMID has been granted orphan drug status in Europe for multiple myeloma and erythema nodosum leprosum [Evaluate, http://www.evaluategroup.com/Universal/View.aspx?type=Story&id=20950, last accessed Nov. 4, 2016]; RAVICTI has been designated orphan medicinal product in the European Union for the treatment of urea cycle disorders (UCDs), and accordingly will benefit from a period of 10 years of regulatory data/market protection with a possibility of extension to 11 years and a period of 10 years of orphan market exclusivity concurrently applied to each of the approved six sub-types of the UCDs [Horizon Pharma, http://ir.horizon-pharma.com/releasedetail.cfm?releaseid=944591, last accessed Nov. 4, 2016].

^38^ Justice Stratas made reference to the following examples of less restrictive legislation in *Takeda, supra* note 16, at 85: The Drug Price Competition and Patent Term Restoration Act, as amended by Pub. L. No. 110-85, 121 Stat. 823 (2007) (of the United States); Regulation 726/2004 (of the European Union); Therapeutic Goods Act, 1989, No. 21 (1990) (of Australia); and the Medicines Act, 1981, No. 118 (of New Zealand). Australia's Federal Court of Appeal has expanded the scope of Section 25A somewhat, in deciding that data protection does not apply where the *active component* of the medicine has previously been included in a registered medicine (including in the form of an enantiomer) [*see Alphapharm Pty Ltd v. H Lundbeck A/S*, [2008] F.C.A. 559].

^39^ *Teva Canada Limited v. Canada (Health)*, 2012 F.C.A. 106.

^40^ *Hospira, supra* note 24.

^41^ *Takeda Canada Inc. v. Minister of Health, et al*., 2013 CanLII 33948 (S.C.C.).

^42^ *Food and Drugs Act*, R.S.C. 1985, c. F-27.

^43^ *Food and Drug Regulations*, *supra* note 5, at C.08.004.1(1).

^44^ Therapeutic Goods Act 1989, at § 25A (Austl).

^45^ Medicines Act 1981, §§ 23A and 23B (NZ).

^46^ 21 U.S.C. at § 355.

^47^ 21 C.F.R. at § 314.108(a).

^48^ The Supreme Court decided that the standard of review is reasonableness for the legislative interpretations of administrative decision makers in *Alberta (Information and Privacy Commissioner) v. Alberta Teachers' Association*, 2011 S.C.C. 61 (CanLII), [2011] 3 S.C.R. 654 at para. 34.

^49^ *Publication of Clarifications to the Guidance Document: Data Protection Under C.08.004.1 of the Food and Drug Regulations*, Health Canada (Oct. 2011), http://www.hc-sc.gc.ca/dhp-mps/alt_formats/pdf/prodpharma/applic-demande/guide-ld/data_donnees_protection-eng.pdf (last accessed Nov. 4, 2016).

^50^ *See, e.g*., *Teva Canada Limited v. Canada (Health)*, 2011 F.C. 507; *Celgene*, FC, *supra* note 14; *Canadian Generic Pharmaceutical Association v. Canada (Health)*, 2011 F.C. 465.

^51^ *Publication of Clarifications to the Guidance Document*, *supra* note 49, at i.

^52^ *See Canadian Generic Pharmaceutical Association v. Canada (Health)*, 2009 F.C. 725 (valid under Trade and Commerce power); *Apotex Inc. v. Canada (Health)*, 2010 F.C.A. 334 (CanLII) (valid under Criminal Law power, and possibly other federal powers). In July 2011, the Supreme Court of Canada dismissed two related applications for leave to appeal [*Apotex Inc. v. Minister of Health et al.*, 2011 CanLII 43416 (S.C.C.); *Canadian Generic Pharmaceutical Association v. Attorney General of Canada et al.*, 2011 CanLII 43415 (S.C.C.)].

^53^ *See* New Zealand Department of Foreign Affairs and Trade, *Text of the Trans-Pacific Partnership*, Trans-Pacific Partnership, arts. 18.50 and 18.52, http://tpp.mfat.govt.nz/text (last accessed Nov. 4, 2016); and *Consolidated CETA Text*, Government of Canada, c. 22, art. 10, http://www.international.gc.ca/trade-commerce/trade-agreements-accords-commerciaux/agr-acc/ceta-aecg/text-texte/consolidated-consolide/toc-tdm.aspx?lang=eng (last accessed Nov. 4, 2016).

